# Supplementary material for: Dynamic transcriptomic profiles of zebrafish gills in response to zinc supplementation
Source: BMC Genomics. 2010 Oct 11;11:553. doi: 10.1186/1471-2164-11-553 (PMC3091702; doi:10.1186/1471-2164-11-553)
Supplement: Additional file 2 — Interactive Direct Interaction Network representing the molecular interactions between zinc, copper, iron, calcium and proteins encoded by transcripts changed by zinc supplementation. Mini web-site containing index.html and hyperlinked pages in subdirectory describing a Direct Interaction Network automatically generated based on curated interactions contained within the proprietary PathwayArchitect database. Ovals represent proteins and the circles symbolize metal ions. Objects are coloured by their abundance in zebrafish at the time-point they were significantly different from the control is a scale from -4 fold (dark green) to +4 fold (dark red). Where significant differences were found at more than one time-point, the colour overlay shows expression at the first instance. Dark blue squares denote 'binding', and light blue squares 'expression'; green squares stand for 'regulation', green diamonds for 'metabolism', and green circles for 'promoter binding'. Arrow heads indicate directionality of the interaction where annotated. All nodes and edges can be further interrogated by selecting the relative area of the image. [file 1471-2164-11-553-S2.zip › PathwayArchitect Zn xs DIN/404244.html]

# TRANSPORT:

|  |  |
| --- | --- |
| Type | TRANSPORT |
| Effect | None |


---

|  |  |
| --- | --- |
| Score | 0 |


---

|  |  |
| --- | --- |
| Reference Count | 5 |


---

|  |  |
| --- | --- |
| Mechanism | Unknown |


---

|  |  |
| --- | --- |
| Reference:0 || Sentence | "From anterior hypothalamic slices cultured in standard medium (i.e., containing adrenal steroids at a final dilution of 61 +/- 9 ng/ml), (a) the stimulatory effect of NA on CRH release was reversed in a dose-dependent manner by increasing concentrations of the alpha1-adrenoreceptor antagonist prazosin, (b) activation of protein kinase C by acute treatment with phorbol 12-myristate 13-acetate (0.5 microM, 1 h) mimicked NA stimulation of CRH secretion, and (c) the activation of L-type Ca2+ channels by Bay K 8644 also produce an increased CRH secretion." |
| PMID | 9489742 |
| Year | 1998 |
| Species | Rat |
| Journal | J Neurochem |
| RefScore | 2 |
| Source | PArchNLP |
  |
|  |  |


---

|  |  |
| --- | --- |
 Reference:1 || Sentence | "The inhibitors of arachidonic acid metabolism, indomethacin, eicosatetraynoic acid, and the calcium channel blocker verapamil, inhibited PAF-stimulated CRH secretion, suggesting mediation by Ca2+ influx and phospholipase-A2 activation." |
| PMID | 2546734 |
| Year | 1989 |
| Species | Rat |
| Journal | Endocrinology |
| RefScore | 1 |
| Source | PArchNLP |
  ||  |  |


---

|  |  |
| --- | --- |
 Reference:2 || Sentence | "The synaptic origin of CRF-IR release in the amygdala was determined in vitro by assessing the Ca2+ dependency of 4-aminopyridine stimulated CRF-IR release from tissue preparations of rat amygdala." |
| PMID | 7643193 |
| Year | 1995 |
| Species | Rat |
| Journal | J Neurosci |
| RefScore | 1 |
| Source | PArchNLP |
  ||  |  |


---

|  |  |
| --- | --- |
 Reference:3 || Sentence | "The increase in CRF secretion at low glucose (0.55 or 1.38 mM) was Ca2+ dependent and completely reversible." |
| PMID | 2901813 |
| Year | 1988 |
| Species | Rat |
| Journal | Am J Physiol |
| RefScore | 0 |
| Source | PArchNLP |
  ||  |  |


---

|  |  |
| --- | --- |
 Reference:4 || Sentence | "Our characterization of the physiologic conditions of KCl-induced release of neuronal CRF using the microdialysis technique provides evidence that the CRF release system is Ca(2+)-dependent and maintains its integrity over many hours in anesthetized rats." |
| PMID | 8047294 |
| Year | 1994 |
| Species | Rat |
| Journal | Neurosci Lett |
| RefScore | 0 |
| Source | PArchNLP |
  |


---

|  |  |
| --- | --- |
